# Early Dual-Antiplatelet Therapy at the Emergency Department Is Associated with Lower In-Hospital Major Adverse Cardiac Event Risk among Patients with Non-ST-Elevation Myocardial Infarction

**DOI:** 10.1155/2021/5571822

**Published:** 2021-04-22

**Authors:** Jen-Han Yang, Hong-Mo Shih, Yan-Cheng Pan, Shih-Sheng Chang, Chi-Yuan Li, Shao-Hua Yu

**Affiliations:** ^1^School of Medicine, College of Medicine, China Medical University, Taichung, Taiwan; ^2^Department of Emergency Medicine, China Medical University Hospital, Taichung, Taiwan; ^3^Division of Cardiovascular Medicine, Department of Internal Medicine, China Medical University Hospital, Taichung, Taiwan; ^4^Department of Anesthesiology, China Medical University Hospital, Taichung, Taiwan; ^5^Graduate Institute of Biomedical Sciences, China Medical University, Taichung, Taiwan

## Abstract

**Background:**

Dual antiplatelet therapy (DAPT) is a standard treatment in non-ST-segment-elevation myocardial infarction (NSTEMI). However, the timing of initiation of DAPT in the Emergency Department (ED) is not well established. The purpose of this study is to demonstrate the correlation between the different timings of DAPT initiation in ED and the outcomes in patients with NSTEMI.

**Method:**

We retrospectively collected data of patients who were diagnosed as NSTEMI in the ED of China Medical University Hospital during 2016 to 2019. All NSTEMI patients who required coronary stenting or ballooning were enrolled into the study, which means NSTEMI patients who received percutaneous coronary intervention (PCI) were included. The time interval between ED arrival and DAPT given was recorded. Patients were divided into 2 groups according to whether they received DAPT within 6 hours after arrival to the ED. The primary outcomes were in-hospital major adverse cardiovascular events (MACE). The secondary outcomes were unexpected return to the ED within 72 hours, readmission within 14 days, and revascularization procedures performed within the first 30 days.

**Results:**

938 NSTEMI patients with PCI were enrolled. Patients who received DAPT beyond 6 hours were relatively old (65.70 ± 14.13 versus 63.16 ± 13.31, *p*=0.014) and had relatively more comorbidities and higher Killip scores than those who received DAPT within 6 hours. The group that received DAPT within 6 hours had lower in-hospital MACE rate (3.52% versus 8.37%, *p*=0.009). Multivariate logistic regression showed the group beyond 6 hours was independently associated with higher risk for in-hospital MACE rate (OR : 2.09, 95% CI 1.07–4.07, *p*=0.030).

**Conclusion:**

Among patients with NSTEMI, DAPT beyond 6 hours after ED arrival have higher in-hospital MACE rate than those within 6 hours.

## 1. Introduction

Non-ST-elevation myocardial infarction (NSTEMI) is a prevalent disease world widely that continues to cause high mortality despite percutaneous coronary intervention (PCI) and improved medication [1, 2]. According to a previous study [3], incidence rates for acute myocardial infraction (AMI), including NSTEMI and ST-elevation myocardial infarction (STEMI), decreased after 2000. However, an epidemiological study using the Taiwan National Health Insurance Research Database revealed that the incidence of NSTEMI has increased progressively in Taiwan, from 11 cases per 100,000 person-years to 28 cases per 100,000 person-years [4]. Fortunately, the in-hospital mortality rate of NSTEMI decreased from 12.2% in 1997 to 7.2% in 2011 [5].

Dual antiplatelet therapy (DAPT) in the treatment of NSTEMI occupies a vital position and has been proven to improve the prognosis of patients with NSTEMI [6–8]. DAPT specifically refers to the combination of antiplatelet therapy with aspirin and a P2Y_12_ receptor inhibitor (clopidogrel, prasugrel, or ticagrelor).

The ESC 2015 guidelines and the AHA/ACC 2014 NSTEMI guidelines suggested that DAPT treatment should begin as soon as possible after PCI. The duration of DAPT has been widely discussed; however, the golden time to initiate DAPT in patients with NSTEMI at the emergency department (ED) is not well established [9]. The AHA/ACC 2014 NSTEMI guidelines [10] recommend the administration of P2Y_12_ inhibitors “before the PCI procedure” without explicitly commenting on when that should be. The ESC 2015 guidelines for NSTEMI patients [11] lack specific recommendations regarding pretreatment. Instead, they state that “the optimal timing of ticagrelor or clopidogrel administration in NSTEMI patients scheduled for an invasive strategy has not been adequately investigated” and that “no recommendation for or against pretreatment with these agents can be formulated.”

The correlations between different timings of the DAPT and the prognosis in patients with NSTEMI remained inconclusive. The objective of this study is to demonstrate whether early administration of DAPT before PCI at the ED could have better outcomes among patients with NSTEMI.

## 2. Methods

### 2.1. Study Design and Population

This retrospective study collected data of patients with NSTEMI admitted to the ED of China Medical University Hospital (CMUH), Taichung, Taiwan, from January 2016 to December 2019. CMUH is a tertiary center in Taiwan with a monthly ED capacity of 14,000 patients, with nearly 650 acute myocardial infarction cases admitted to the ED annually. Nearly 550 cases of coronary catheterization and 450 cases of percutaneous coronary intervention were performed in AMI patients from the ED annually.

Patients with acute myocardial infarction were enrolled into the study initially. Patients with acute coronary syndrome (ACS) with electrocardiography (ECG) ST segment elevation were excluded. Only patients with NSTEMI, which was defined as elevated cardiac enzymes without electrocardiography (ECG) ST segment elevation, were enrolled in this study. Patients were also excluded if they did not complete DAPT in the ED for any reason. Patients with NSTEMI who did not receive coronary angiography were excluded. Those who underwent coronary angiography without significant coronary artery stenosis were also excluded. Only patients with NSTEMI who had significant coronary artery stenosis from angiography that require PCI, including stenting or balloon dilatation, were enrolled into the study.

### 2.2. Data Collection

We recorded the time interval between the time the patient arrived at the ED and the time that DAPT was initiated. Initiation of DAPT was defined as aspirin and a P2Y_12_ inhibitor (clopidogrel, prasugrel, or ticagrelor) first given in the ED with a pharmacological-suggested loading dose. Patient demographic data, including sex, age, and underlying diseases such as smoking history, hypertension, diabetes mellitus, coronary artery disease, cerebrovascular disease, chronic kidney disease, and hyperlipidemia, were also recorded. Furthermore, the presentation features such as blood pressure, heart rates, Killip scores, and cardiac biomarkers obtained in the ED and initial cardiac catherization results were also collected.

This study was approved by the Institutional Review Board of China Medical University. Written informed consent was not obtained from the patients because of the retrospective nature of the study.

### 2.3. Outcome Measurement

The primary outcomes were patient in-hospital major adverse cardiovascular events (MACE) (including stroke, recurrent myocardial infarction, rupture PCI, and death) and cardiogenic shock. The secondary outcomes were returned to the ED within 72 hours or readmitted in 14 days, and revascularization procedures were performed within the first 30 days.

### 2.4. Statistical Analysis

The patients were divided into 2 groups. The “early DAPT group” was defined as the time interval between ED arrival and initiation of DAPT less than 6 hours, and the “late DAPT group” was defined as the time interval more than 6 hours. Patient characteristics, preexisting comorbidities, and presentation features of these 2 groups were recorded. The primary outcomes and secondary outcomes in the 2 groups were compared.

The categorical variables were presented as percentages and examined using chi-square tests. The continuous variables were presented as the mean ± standard deviation and analyzed using independent-sample *t*-tests.

Furthermore, univariate analysis was used to identify the factors associated with MACE. Significant and important variables were entered into a stepwise backward logistic regression analysis. All statistical analyses were performed using SAS software version 9.4 (SAS Institute Inc., Cary, NC, USA). A two-tailed *p* value of less than 0.05 was considered significant.

## 3. Results

**Figure 1:**
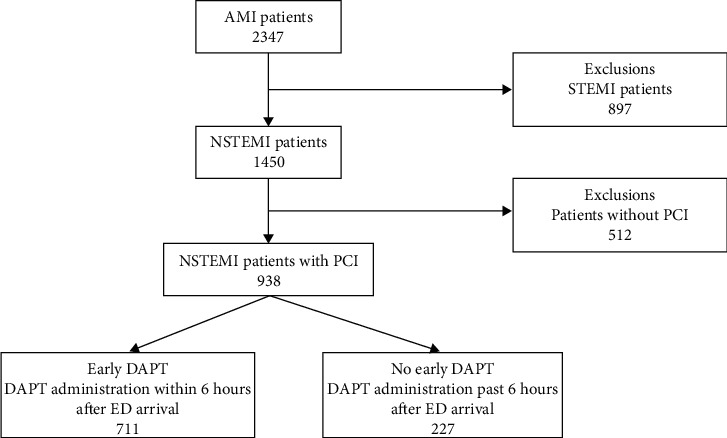
Flow chart of patient enrollment.

A total of 2347 patients were diagnosed as AMI during the study period, and 897 patients were diagnosed as STEMI. 1450 NSTEMI patients were diagnosed. Among these patients, 938 patients received percutaneous coronary intervention and were enrolled int this study. 711 patients with NSTEMI were given DAPT within 6 hours (early DAPT group), and 227 patients were given DAPT beyond 6 hours (late DAPT group) (shown in Figure 1).

AMI indicates acute myocardial infarction; STEMI, ST-elevation myocardial infarction; NSTEMI, non-ST-elevation myocardial infarction; PCI, percutaneous coronary intervention; and DAPT, dual antiplatelet therapy.

Baseline characteristics are presented in Table 1. The “late DAPT group” was older (65.70 ± 14.13 versus 63.16 ± 13.31, *p*=0.014). Men were dominant in both groups, with the higher percentage in the “early DAPT group” (76.37% versus 65.64%, *p*=0.001). The “late DAPT group” had higher prevalence of underlying disease such as diabetes mellitus (54.19% versus 41.07%, *p* < 0.001), chronic kidney disease (34.36% versus 21.66%, *p* < 0.001), and hyperlipidemia (11.45% versus 6.89%).

Patients in the “late DAPT group” had higher Killip score (II to IV) (19.13% versus 27.31%, *p*=0.018). There was no significant difference in other presentation features or angiographic findings in left main or multivessel coronary disease.

The patients who received DAPT within 6 hours (early DAPT group) showed better outcome in in-hospital MACE rate than the patients who received DAPT past 6 hours (late DAPT group) (3.52% versus 8.37%, *p*=0.009). The in-hospital mortality rate was lower in the early DAPT group than the late DAPT group (2.95% versus 7.05%, *p*=0.009). Although there was no statistical significance, there were less in-hospital stroke and recurrent myocardial infarction events in the early DAPT group. There was no significant difference in the secondary outcomes such as 72-hour return to ED after discharge, readmission within 14 days after discharge, PCI performed within 30 days, or CABG performed within 30 days in both groups (Table 2).

Age, timing of DAPT given, prior cerebrovascular disease (CVA), higher Killip score, and peak troponin I levels were associated with increased MACE rate in univariate analysis. Significant variables (age, timing, prior CVA, Killip score, and peak troponin I levels) and several important variables (sex, heart rates, smoking, DM, and CKD) were entered into a stepwise backward logistic regression analysis. Further multivariant analysis by adjust demonstrated that elder patients had higher odds ratio risk for in-hospital MACE rate (OR : 1.05, 95% CI 1.02–1.08, *p* < 0.001). Patients who received DAPT past 6 hours were independently associated with higher risk for in-hospital MACE (OR : 2.09, 95% CI 1.07–4.07, *p*=0.03). Patients who had higher Killip scores II to IV and higher peak troponin I at ED presentation also had higher risk for in-hospital MACE (OR : 5.16, 95% CI 2.65–10.03, *p* < 0.001, OR : 1.02, 95% CI 1.01–1.04, *p*=0.048, respectively) (Table 3).

## 4. Discussion

Our study pointed out that, among patients with NSTEMI who need percutaneous coronary intervention, as patients of type I MI, which defined as MI related to atherosclerotic plaque rupture, ulceration, fissuring, erosion, or dissection with intraluminal thrombus in one or more of the coronary arteries, leading to decreased myocardial blood flow or distal platelet emboli and, thereby, resulting in myocyte necrosis [12]. Patients who received DAPT past 6 hours after arrival to the emergency department have higher in-hospital MACE rate 2-fold greater than those who received DAPT within 6 hours. The elderly also had greater in-hospital MACE rate in NSTEMI with type I MI. Not surprisingly, when patients with NSTEMI presented to the ED, higher Killip scores and higher peak troponin I levels also had higher in-hospital MACE rates. This study was specified on the patient with NSTEMI requiring coronary stenting or ballooning. Also, the study focused on the effect of very early DAPT within 6 hours after medical contact.

DAPT with aspirin and a P2Y_12_ receptor inhibitor can reduce the risk of ischemic events. The outcomes in patients with NSTEMI were affected by the timing of PCI and DAPT duration after PCI [8]. However, the relationship of the patient outcomes and pretreatment of DAPT, which means DAPT before PCI, still remains controversial. It is logical to assume that early administration of DAPT prior to coronary angiography (referred to as upstream therapy or pretreatment) and PCI should provide greater benefit [8, 9]. However, pros and cons of DAPT pretreatment in NSTEMI patients were debating. Pros of early DAPT before PCI suggest that achieving maximal platelet inhibition early in the clinical presentation may be beneficial in reducing infarction size and may lower the risk of stent thrombosis for patients undergoing percutaneous revascularization, thereby preventing downstream morbidity. Several guideline recommendations have suggested for DAPT administration early in hospital course [10, 13]. However, inhibiting platelets prior to an invasive procedure could increase the risk of bleeding [14–16]. The discussed disadvantages of DAPT before PCI include higher procedural bleeding risk, delays in patients requiring surgical intervention, and higher risk of coronary-artery-bypass-graft- (CABG-) related bleeding if surgical anatomy is found and emergency surgery is required [17–19]. Concerns for increases in bleeding or delay in revascularization if P2Y_12_ receptor inhibitors needed to be eliminated from circulation among patients requiring surgical revascularization [14, 20]. Due to these pros and cons of data, current guidelines are no longer able to advocate for or against pretreatment with these agents [21].

A previous study pointed out that, in NSTEMI patients, administration of DAPT within 24 hours was not associated with any improvement in long-term outcomes [22]. There were several differences between previous studies and our study. First, we enrolled the study population within NSTEMI with PCI to exclude type 2 myocardial infarction since the cause of ischemia in these groups is not related to occlusion of the coronary artery and may relate to hypovolemia, sepsis, or end-stage renal disease. DAPT does not have any effect on type 2 myocardial injury. We also set up time differences to within or beyond 6 hours to evaluate the very-early-administrated DAPT effectiveness. To our knowledge, this is the first study to evaluate the outcome of early DAPT within 6 hours in patients with NSTEMI of type 1 MI, and our study results demonstrated that the patients with NSTEMI who received DAPT beyond 6 hours had higher in-hospital MACE rate than those within 6 hours.

Our study showed that NSTEMI patients with PCI who received DAPT beyond 6 hours were older, having more comorbidities such as diabetes mellitus, chronic kidney disease, and hyperlipidemia and had higher Killip scores. Previous studies [22, 23] demonstrated that DAPT was postponed or cancelled due to the patients with older age and multiple comorbidities. These results may relate to unspecific symptoms or signs, concerning about high-risk factors such as bleeding in these groups of NSTEMI patients. However, after adjusted with multivariate logistic regression, the early DAPT group remained to have a lower risk of MACE rate independently in this study.

### 4.1. Limitations

The present study has the following limitations. First, being a single-center retrospective observational study, the risk of selection bias is present. Since the study population was relatively small, the statistical analysis has an inherent risk of beta error. Furthermore, we could not gather information about symptom onset time or signs, and the exact NSTEMI onset time was unknown. Given the time limitation, we could not calculate one-year mortality rate for outcome evaluation for each group. Additional prospective studies are warranted to provide information on potential improvement of the AMI network.

## 5. Conclusions

Our study demonstrated that NSTEMI patients with DAPT delayed by more than 6 hours after ED arrival have higher in-hospital MACE rate than those within 6 hours.

## Figures and Tables

**Table 1:** Patient characteristics.

Variables	Time		*p* value
	<6 hours (*n* = 711)	>6 hours (*n* = 227)	
Demographics			
Age, mean ± SD	63.16 ± 13.31	65.70 ± 14.13	0.014^a^

Sex, (%)			0.001^b^
Male	543 (76.37)	149 (65.64)	
Female	168 (23.63)	78 (34.36)	

Clinical diseases history, (%)			
Smoking	356 (50.07)	97 (42.73)	0.054^b^
Hypertension	436 (61.32)	152 (66.96)	0.126^b^
Diabetes mellitus	292 (41.07)	123 (54.19)	<0.001^b^
Coronary artery disease	208 (29.25)	76 (33.48)	0.227^b^
Cerebrovascular disease	43 (6.05)	13 (5.73)	0.859^b^
Chronic kidney disease	154 (21.66)	78 (34.36)	<0.001^b^
Hyperlipidemia	49 (6.89)	26 (11.45)	0.027^b^
Presentation features			
Killip class II–IV, %	19.13	27.31	0.018
Systolic BP	140.0 ± 32.87	139.9 ± 39.72	0.975^a^
Diastolic BP	85.96 ± 23.07	83.29 ± 24.39	0.137^a^
Heart rate, bpm	87.49 ± 22.71	91.34 ± 27.10	0.056^a^
Peak troponin I, ng/mL	5.82 ± 11.43	6.24 ± 13.78	0.685^a^

Angiographic findings			
Left main disease ^*∗*^	67 (9.42)	18 (7.93)	0.494^b^
No. of disease vessels ^*∗*^			0.056^b^
1	311 (43.74)	109 (48.02)	
2	229 (32.21)	75 (33.04)	
3	130 (18.28)	25 (11.01)	

Data are presented as mean ± SD for continuous variables and number (percentage) for categorical variables. a: two-sample *T* test, b: chi-square test. BP indicates blood pressure,  ^*∗*^Significant disease is stenosis >75% in 1 coronary artery, except left main disease (stenosis >50%).

**Table 2:** Patient outcome in each group.

Variable	Time		*p* value
	<6 hours (*n* = 711)	>6 hours (*n* = 227)	
In-hospital MACE, (%)			0.009
No	686 (96.48)	208 (91.63)	
Stroke	3 (0.42)	2 (0.88)	
Recurrent MI	0 (0.00)	1 (0.44)	
Rupture PCI	1 (0.14)	0 (0.00)	
Death	21 (2.95)	16 (7.05)	
	25 (3.52)	19 (8.37)	

72 hours ED return (%)			0.095
No	705 (99.16)	222 (97.80)	
Planned	2 (0.28)	3 (1.32)	
Unplanned	4 (0.56)	2 (0.88)	

14 days readmission (%)			0.144
No	693 (97.47)	216 (95.15)	
Planned	10 (1.41)	5 (2.20)	
Unplanned	8 (1.13)	6 (2.64)	
PCI performed within 30 days	90 (12.66)	18 (7.93)	0.052^b^
CABG performed within 30 days	9 (1.27)	0 (0.00)	0.123^b^

a: two-sample *T* test. b: chi-square test. MACE indicates major adverse cardiovascular disease; MI, myocardial infarction; PCI, percutaneous coronary intervention; ED, emergency department; CABG, coronary artery bypass graft.

**Table 3:** Univariate and multivariate analysis for in-hospital MACE rates.

Parameters	Univariate		Multivariate	
	OR (95% CI)	*p* value	OR (95% CI)	*p* value
Demographics				
Age	1.06 (1.03–1.08)	<0.001	1.05 (1.02–1.08)	<0.001

Sex				
Female	Ref.	—	Ref.	—
Male	0.95 (0.48–1.87)	0.871	1.56 (0.71–3.45)	0.271

Time				
<6 hours	Ref.	—	Ref.	—
>6 hours	2.51 (1.35–4.64)	0.003	2.09 (1.07–4.07)	0.030

Clinical diseases history				
Smoking	0.73 (0.40–1.35)	0.317	0.88 (0.42–1.83)	0.731
Hypertension	1.83 (0.91–3.67)	0.087	—	—
Diabetes mellitus	1.05 (0.57–1.93)	0.868	0.71 (0.36–1.40)	0.322
CAD	0.96 (0.50–1.87)	0.914	—	—
CVA	2.67 (1.08–6.60)	0.034	1.64 (0.60–4.47)	0.332
CKD	1.02 (0.51–2.04)	0.966	0.68 (0.30–1.51)	0.342
Hyperlipidemia	0.84 (0.25–2.77)	0.768	—	—

Presentation features				
Killip class				
Level 1	Ref.		Ref.	
Level 2–level 4	5.48 (2.95–10.19)	<0.001	5.16 (2.65–10.03)	<0.001
Heart rates	0.99 (0.98–1.01)	0.596	0.99 (0.98–1.00)	0.137
Peak troponin I	1.02 (1.01–1.04)	0.009	1.02 (1.01–1.04)	0.048

Angiographic finding				
Left main disease	1.31 (0.50–3.40)	0.586	—	—
No. of disease vessels				
1	3.52 (0.47–26.48)	0.150	—	—
2	2.18 (0.28–17.19)	0.959	—	—
3	3.16 (0.39–25.80)	0.355	—	—

CAD, coronary artery disease; CVA, cerebrovascular disease; CKD, chronic kidney disease.

## Data Availability

The dataset used to support the findings of this study is restricted by the China Medical University and Hospital Research Ethics Committee in order to protect patient privacy. Data are available from the corresponding author for researchers who meet the criteria for access to confidential data.

## Abbreviations

NSTEMI: Non-ST-segment-elevation myocardial infarction
PCI: Percutaneous coronary intervention
AMI: Acute myocardial infarction
STEMI: ST-segment-elevation myocardial infarction
DAPT: Dual antiplatelet treatment
ED: Emergency department
ACS: Acute coronary syndrome
ECG: Electrocardiograph
MACE: Major adverse cardiovascular event
CABG: Coronary artery bypass graft
CVA: Cerebrovascular accident
BP: Blood pressure.
